# Socket Array Irregularities and Wing Membrane Distortions at the Eyespot Foci of Butterfly Wings Suggest Mechanical Signals for Color Pattern Determination

**DOI:** 10.3390/insects15070535

**Published:** 2024-07-16

**Authors:** Yugo Nakazato, Joji M. Otaki

**Affiliations:** The BCPH Unit of Molecular Physiology, Department of Chemistry, Biology and Marine Science, Faculty of Science, University of the Ryukyus, Nishihara 903-0213, Okinawa, Japan

**Keywords:** butterfly wing, eyespot, mechanical signal, organizer, color pattern, scale, socket, scanning electron microscopy (SEM)

## Abstract

**Simple Summary:**

Eyespot foci on butterfly wings function as organizers for eyespot color patterns during development. Here, we microscopically examined the scales, sockets, and wing membrane at the eyespot foci of butterfly wings using the Blue Pansy butterfly. Although not always, at the eyespot foci, scales showed disordered planar polarity, sockets were irregularly positioned, and the wing membrane was physically distorted. Physical damage in the background area induced ectopic patterns with socket array irregularities and wing membrane distortions, similar to natural eyespot foci. These results suggest that either the process of determining an eyespot focus or the function of an eyespot organizer may be associated with wing-wide mechanics that physically disrupt socket cells, scale cells, and the wing membrane.

**Abstract:**

Eyespot foci on butterfly wings function as organizers of eyespot color patterns during development. Despite their importance, focal structures have not been examined in detail. Here, we microscopically examined scales, sockets, and the wing membrane in the butterfly eyespot foci of both expanded and unexpanded wings using the Blue Pansy butterfly *Junonia orithya*. Images from a high-resolution light microscope revealed that, although not always, eyespot foci had scales with disordered planar polarity. Scanning electron microscopy (SEM) images after scale removal revealed that the sockets were irregularly positioned and that the wing membrane was physically distorted as if the focal site were mechanically squeezed from the surroundings. Focal areas without eyespots also had socket array irregularities, but less frequently and less severely. Physical damage in the background area induced ectopic patterns with socket array irregularities and wing membrane distortions, similar to natural eyespot foci. These results suggest that either the process of determining an eyespot focus or the function of an eyespot organizer may be associated with wing-wide mechanics that physically disrupt socket cells, scale cells, and the wing membrane, supporting the physical distortion hypothesis of the induction model for color pattern determination in butterfly wings.

## 1. Introduction

The color patterns of butterfly wings are highly diverse, but they are believed to be derived from various modifications of a general wing-wide pattern called the nymphalid groundplan [1,2,3,4,5,6]. The nymphalid groundplan is composed of a collection of color pattern elements positioned on a white background (i.e., on wings without color patterns). One of the most conspicuous color pattern elements is a border ocellus (i.e., eyespot) in the border symmetry system. Some nymphalid butterflies, such as the Squinting Bush Brown *Bicyclus anynana* in Africa, the Common Buckeye *Junonia coenia* in America, and the Blue Pansy *Junonia orithya* in Asia, have large eyespots on their wings, and they have been used for studying the developmental mechanisms of eyespot pattern formation. Using *J. coenia*, Nijhout (1980) [7] elegantly demonstrated that the prospective focus of an eyespot in pupal wing tissue functions as an organizing center for eyespot color pattern formation via cautery (physical damage) and transplantation experiments. Moreover, Nijhout (1985) [8] showed that ectopic eyespots can be induced by cautery in the background area of the wings. Other groups independently confirmed these results [9,10,11,12,13,14,15,16]. Since then, how an eyespot is determined during development has attracted intensive research interest in the fields of insect physiology, developmental biology, and evolutionary biology. Accordingly, many studies have investigated genes expressed at eyespot foci and other elements [17,18,19,20,21,22,23,24,25,26,27,28,29,30,31,32,33,34,35,36,37,38,39,40,41,42,43,44,45,46,47,48]. However, how molecules and cells behave to determine eyespot color patterns remains elusive.

Moreover, information at the cellular and tissue levels on eyespot foci has gradually accumulated. Bioimaging studies have successfully recorded live wings [49,50,51] and eyespot focal cells in vivo [52]. Morphometric studies revealed that scales are larger at eyespot foci than in surroundings [53,54]. The pupal wing surface has pupal cuticle focal spots just above the organizing cells, causing physical distortion of the pupal wing tissue [13]. The eyespots of adult wings are also distorted [55]. There is a positive correlation between pupal cuticle focal spot size and adult eyespot size in *Junonia* [55]. These studies suggest that the pupal cuticle focal spot is important for eyespot color pattern formation. Based on color pattern analyses of various nymphalid butterflies, the induction model for color pattern determination has been proposed [56,57,58]. In this model, a morphogenic signal for color pattern determination is a serial and slow wave-like signal that follows Newtonian motion [59]. The physical distortion hypothesis was then introduced to explain the mechanical signal for color pattern determination [58]. This hypothesis states that important morphogenic signals are provided in the form of wave-like physical distortions of an epithelial sheet instead of molecules [58] and was shown not to contradict real butterfly eyespots in terms of color gradients and other features [60].

From a physiological (or pharmacological) perspective, color patterns can be experimentally modified by cold shock treatments of pupae [1,61,62] as well as by injecting specific chemicals (modification inducers) into pupae [63,64,65,66]. These color pattern modifications can be interpreted as modifications of morphogenic signals in pupal wings. The extracellular matrix appears to play a critical role in morphogenic signal transport [67,68]. Modification inducers appear to act on chitin in the cuticle [69]. These results are consistent with the distortion hypothesis and the induction model. However, other possibilities should not be ruled out. For example, cytonemes [70,71,72,73] may play an important role in determining color patterns in butterflies [50].

Additionally, ubiquitous low-molecular-weight molecules such as calcium ions may function as morphogenic signals in butterfly wings and may be able to travel relatively long distances due to their low molecular weight and the amplification of signals to form a wave-like signal [74]. To explain the fact that ectopic eyespots are induced by physical damage to the background area [1,8,10,14,15,16], low-molecular-weight molecules such as calcium ions may be released from the damage site to surrounding cells immediately after damage [74]. In contrast, the immediate synthesis of molecular morphogens at the damaged site may be difficult due to the time limitation of the critical (sensitive) period.

These studies above discuss fate-determining molecules or signals from eyespot focal cells. The products (i.e., scales, sockets, and the wing membrane) that constitute the focal structures in adults may reflect the activity of the focal cells. Nevertheless, despite their importance, the focal structures have not been examined in detail. Here, we used a high-resolution digital light microscope and a scanning electron microscope to microscopically examine the scales, sockets, and wing membrane in the butterfly eyespot foci of both expanded (post-eclosion) and unexpanded (pre-eclosion) wings of the Blue Pansy butterfly *J. orithya*. We looked for evidence of mechanical distortion of eyespot foci according to the distortion hypothesis and the induction model. We also examined the damaged site that functioned as an organizer for ectopic color patterns. Based on the present and previous results, we discussed possible models for color pattern determination in butterfly wings. We also discussed the present results in light of the current status of mechanobiology.

## 2. Materials and Methods

### 2.1. Butterflies and Wing Specimens

Adult females of the Blue Pansy butterfly *Junonia orithya* (Linnaeus, 1758) (Lepidoptera: Nymphalidae: Nymphalinae: Junoniini) were caught on the Nishihara campus of the University of the Ryukyus and its vicinity on Okinawa-jima Island, Japan. Eggs were collected on a natural host plant, *Phyla nodiflora*, within a glass tank in the laboratory, and larvae were reared on two host plant species, *P. nodiflora* and *Plantago asiatica*.

**Figure 1:**
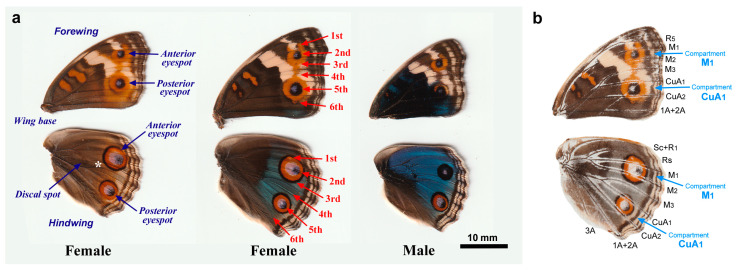
Wing color patterns of the Blue Pansy butterfly *J. orithya*. (**a**) Dorsal wings. Males have a single blue form (right), and females have blue and brown forms (middle and left, respectively). In each wing, there are two eyespots, the anterior and posterior ones. Potential eyespot foci are named the first, second, third, fourth, fifth, and sixth focal areas from the anterior to posterior sides. An asterisk indicates the background area where physical damage was made to induce ectopic color patterns. (**b**) Dorsal cover scale image. Dorsal cover scales were removed at once from the wings with transparent adhesive tape for the purpose of illustrating wing veins and compartments. Scales along the wing veins are not present in this image, highlighting the wing veins. The anterior and posterior eyespot foci are located in the compartments M_1_ and CuA_1_, respectively.

The color patterns of females in *J. orithya* somewhat vary among individuals. There are two major forms, the brown form and the blue form, in females based on the color difference in the background area (Figure 1). Males have only the blue form. We defined “expanded” wings as wings that completely expanded after eclosion outside a pupal case. We defined “unexpanded” wings as the wings immediately before eclosion within a pupal case. Examination of unexpanded wings may help us detect focal disorders due to the high density of scales and sockets and the relatively compact size of the wing for microscopic analyses.

### 2.2. Nomenclature

The nomenclature of eyespots and focal areas is shown in Figure 1a. There are two eyespots on the dorsal surfaces of the wings. These eyespots are called anterior and posterior eyespots. Focal areas are called the first to sixth areas from the anterior to the posterior direction in consecutive wing compartments. The anterior and posterior eyespots correspond to the second and fifth focal areas, respectively. More formally, the anterior and posterior eyespot foci are present in the wing compartments M_1_ and CuA_1_, respectively (Figure 1b). We focused only on the dorsal side of the wings because the dorsal eyespots are larger than the ventral eyespots and because the dorsal color patterns are much simpler than the ventral color patterns. In males, the anterior eyespot in the hindwing is occasionally very small.

### 2.3. Physical Damage

Physical damage was made to induce ectopic color patterns on the dorsal hindwings. Pupae were treated with a Shiga Konchu No. 0 insect pin (Tokyo, Japan), which is a fine stainless needle, two to three hours post-pupation. The diameter of the needle was 0.34 mm. The needle was placed in the background area between the anterior and posterior eyespots (Figure 1a). The needle was moved slowly up and down at the damaged site at a depth of approximately 3 mm. The treated pupae were allowed to develop to the adult stage.

### 2.4. Microscopic Analyses and Sample Preparation

Wing samples were examined using two kinds of microscopes: a Keyence high-resolution Digital Microscope VHX-7000 (Osaka, Japan) for surface light microscopic observation of scales and a Hitachi High-Tech Tabletop Scanning Electron Microscope (SEM) TM3030 or TM4000PlusII (Tokyo, Japan) for the observation of sockets and the wing membrane after the removal of scales. Sockets and the wing membrane after the removal of scales are semitransparent in this species and cannot be observed well with a light microscope. In contrast, SEM images can be obtained that reveal surface microstructures.

For the light microscopic observations, both expanded and unexpanded wings were removed from the thorax before the observations. For the latter, the isolated wings were sandwiched with glass slides on which a weight (a plastic bottle containing more than 200 mL of water) was placed for more than six hours until the unexpanded wings became flat. For SEM observations, the scales of the expanded wings were removed with a fine brush. The scales of the unexpanded wings were removed with adhesive tape. When removing scales with ectopic color patterns, we used double-sided adhesive tape that was placed at the tip of the head of a needle so as not to damage the sockets or the wing membrane.

### 2.5. Definitions, Frequency Calculations, and Statistics

The frequencies of scale arrangement disorder were obtained for the male posterior eyespot, the female anterior eyespot, and the female posterior eyespot. To do so, the scale arrangement disorder was defined to include the following three factors: (1) substantial differences in the directions (planar polarity) of scales; (2) extensive scale overlap; and (3) high scale density. This is a working definition that may suffer from subjective judgments but was tentatively useful to obtain nonrandom samples with the scale arrangement disorder. The socket array irregularity was defined as any irregularity of socket arrays with socket displacement, a long or short socket interval, and an irregular direction (planar polarity) of a socket. The wing membrane distortion was defined as an unusual distortion of the wing membrane associated with multiple sockets but not with just a single socket.

The number of individuals with wing membrane distortion was compared between a random sampling group and a nonrandom sampling group of scale arrangement disorders with Fisher’s exact test using JSTAT (Yokohama, Japan). Random sampling was performed using Microsoft Excel’s random generator function (RAND function) with individual sample numbers from a pool of 60 individuals. To do so, each individual was associated with a number generated with the RAND function, which was then ranked. The top 11 individuals were considered members of the random sampling group and were subjected to SEM observations.

## 3. Results

### 3.1. Eyespot Focal Areas of Expanded Wings

**Figure 2:**
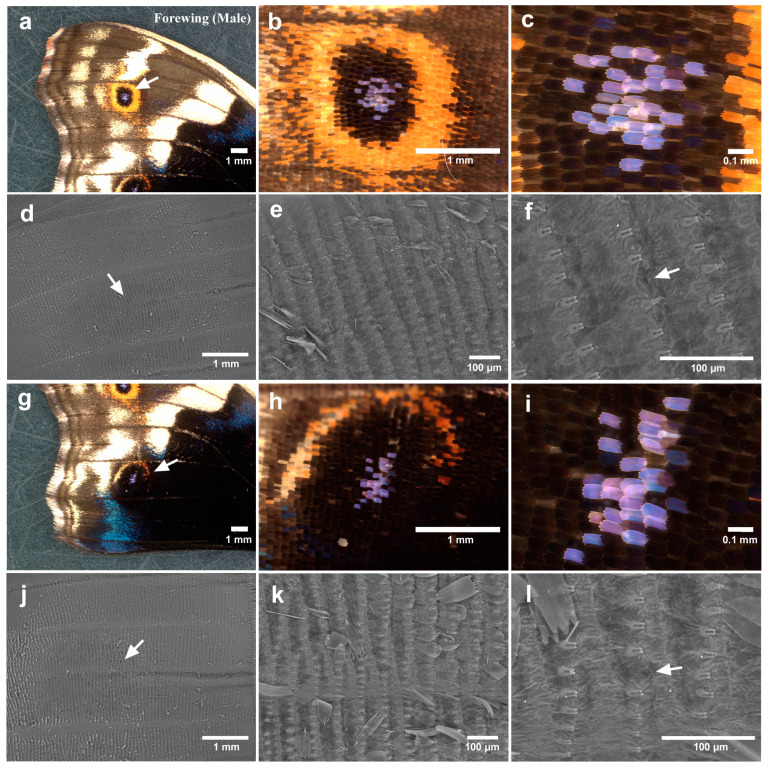
Forewing eyespots. (**a**) The anterior eyespot (an arrow). (**b**) Magnification of the anterior eyespot shown in (**a**). (**c**) Focal area of (**b**). (**d**) Focal area without scales. An arrow indicates the location of the focal area, nearly at the end of the midline. This is a contralateral wing from the wing shown in (**a**–**c**). The following e and f are also contralateral wings. Thus, the wing margin is to the right. (**e**) Magnification of (**d**). (**f**) Magnification of (**e**). An arrow indicates a relatively large socket interval in the focal area. (**g**) The posterior eyespot (an arrow). (**h**) Magnification of the posterior eyespot shown in (**g**). (**i**) Focal area of (**h**). Scale directions appeared to be somewhat irregular. (**j**) Focal area without scales. An arrow indicates the location of the focal area, nearly at the end of the midline. This is a contralateral wing from the wing shown in (**g**–**i**). The following (**k**,**l**) are also contralateral wings. Thus, the wing margin is to the right. (**k**) Magnification of (**j**). (**l**) Magnification of (**k**). An arrow indicates a relatively large socket interval in the focal area.

We first observed the eyespot foci of adult forewings that expanded after eclosion as a normal process of metamorphosis. We first focused on the anterior eyespot (second focal area). No irregularities in scale arrangement or scale size were clearly observed under a light microscope in any of the individuals examined (male *n* = 4; female *n* = 4) (Figure 2a–c). When the scales were removed, we observed via SEM that the eyespot focus was located nearly at the end of the clear midline in all individuals (male *n* = 2; female *n* = 1) (Figure 2d). A midline was imaged as a prominent band or groove (as in other cases throughout this study). Socket arrays appeared to be largely regularly placed but were somewhat irregular at the eyespot foci; socket intervals at the foci seemed to be larger than those of the surroundings in two individuals (Figure 2e,f). One socket might have failed to develop there.

Similar observations of the posterior eyespots of the forewings were made. For light microscopy images, the white or blue structural focal scales overlapped with neighboring scales and were more disordered in terms of the scale direction (i.e., planar polarity) in two individuals among the individuals examined here (male *n* = 4; female *n* = 4) (Figure 2g–l). Notably, white or blue focal scales were not always observed at eyespot foci in this species. In the SEM images, the focus appeared to be located at the end of the midline, and the socket intervals appeared to be larger at the focus in all individuals (male *n* = 2; female *n* = 1).

**Figure 3:**
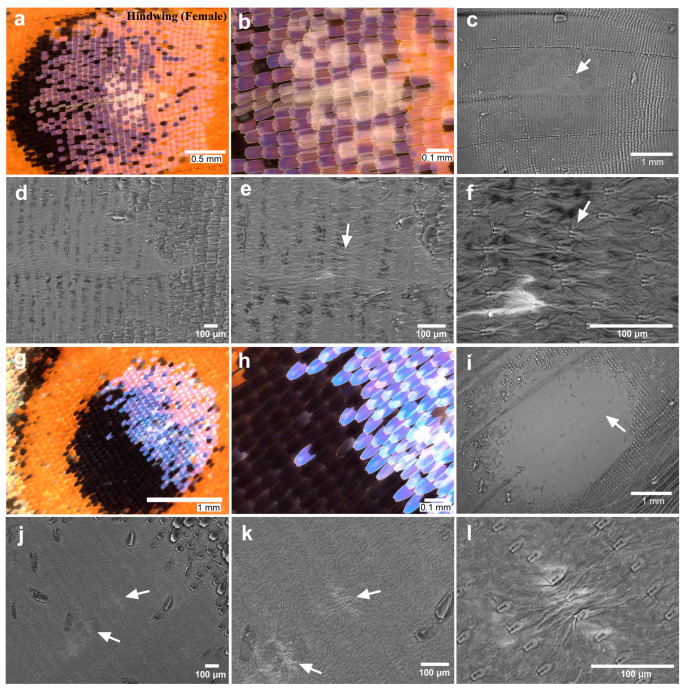
Anterior eyespot in the hindwing (female). (**a**) The anterior eyespot of an individual. (**b**) Magnification of the focal area of the posterior eyespot shown in (**a**). White focal scales are positioned without disorganization. (**c**) The eyespot area without scales. An arrow indicates the focal area at the end of the midline. This eyespot is identical to the one shown in (**a**,**b**). (**d**) Magnification of (**c**). (**e**) Magnification of (**d**). An arrow indicates an area of socket array irregularities. (**f**) Magnification of (**e**). An arrow indicates an area of socket array irregularities. (**g**) The anterior eyespot of another individual. (**h**) Magnification of the focal area of the posterior eyespot shown in (**g**). Blue and white focal scales are positioned without disorganization. (**i**) The eyespot without scales. An arrow indicates the focal area at the end of the midline. This eyespot is identical to the one shown in (**g**,**h**). (**j**) Magnification of (**i**). Arrows indicate distortions of the wing membrane. (**k**) Magnification of (**j**). Arrows indicate distortions of the wing membrane. (**l**) Magnification of (**k**).

We then observed the eyespot foci of the expanded hindwings. We first focused on anterior eyespot foci (second focal area) using a light microscope (male *n* = 20; female *n* = 20). The focal white structural scales appeared to be denser than the surrounding scales, with extensive overlaps (Figure 3a,b). After the removal of the scales for SEM, the anterior eyespot focus seemed to be located nearly at the end of the midline in all the individuals examined (female *n* = 5) (Figure 3c). We found that the focal sockets were irregularly placed in one individual (Figure 3d–f). In another individual, the focal scales did not seem to be disordered in the light microscopy images (Figure 3g,h). However, when scales were removed from this wing for SEM, we detected striking physical distortions of the wing membrane associated with a few scales, as if those distortions were produced as a result of mechanical pushes both from the anterior and posterior sides (Figure 3i–l). Similar wing membrane distortions were observed in all the individuals examined here.

**Figure 4:**
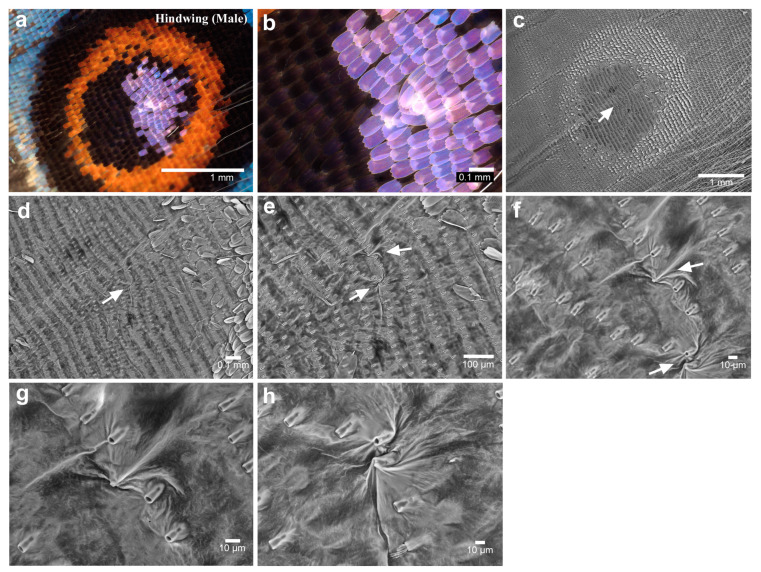
Posterior eyespot in the hindwing (male). (**a**) The posterior eyespot. (**b**) Magnification of the focal area of the posterior eyespot shown in (**a**). White focal scales are disorganized in direction. (**c**) The eyespot area without scales. The arrow indicates the focal area at the end of the midline. This eyespot is identical to the one shown in (**a**,**b**). (**d**) Magnification of (**c**). An arrow indicates an area of disorder. (**e**) Magnification of (**d**). Arrows indicate striking distortions of the wing membrane, one of which is associated with the unusual arrangement of socket arrays. (**f**) Magnification of (**e**). Arrows indicate striking distortions of the basal membrane. (**g**) Magnification of (**f**). (**h**) Another magnification of (**f**).

We next focused on the posterior eyespot foci (fifth focal area) of the samples used for the anterior eyespot foci (male *n* = 20; female *n* = 20). One of the individuals examined showed a disordered scale arrangement at the focus, as clearly observed in the light microscopy images (Figure 4a,b). The directions (planar polarity) of some white focal scales were clearly distorted compared to those of other neighboring scales. No individual was clearer than this individual in terms of scale disorder in the light microscopy images. It appeared that this type of scale disorder varied in degree depending on the individual. However, three additional individuals (four individuals in total) had scale disorders similar to this one but with less intensity. Thus, 10% (4/40) of individuals had the scale arrangement disorder in the posterior eyespot foci among the individuals examined here.

The scales were then removed for SEM from the wings of the individuals showing scale disorders in the anterior eyespots under light microscopic observation (male *n* = 1; female *n* = 2). The anterior eyespot focus seemed to be located nearly at the end of the midline in all individuals examined (Figure 4c). We detected irregularities in socket arrays in the eyespot focal area in one individual (Figure 4c,d). Moreover, irregularities in socket arrays were associated with striking physical distortions of the wing membrane (Figure 4e–h). One additional individual showed similar socket array irregularities and wing membrane disorders with less intensity. No other individuals examined showed similar physical distortions. 

### 3.2. Eyespot Focal Areas of Unexpanded Wings before Eclosion

**Figure 5:**
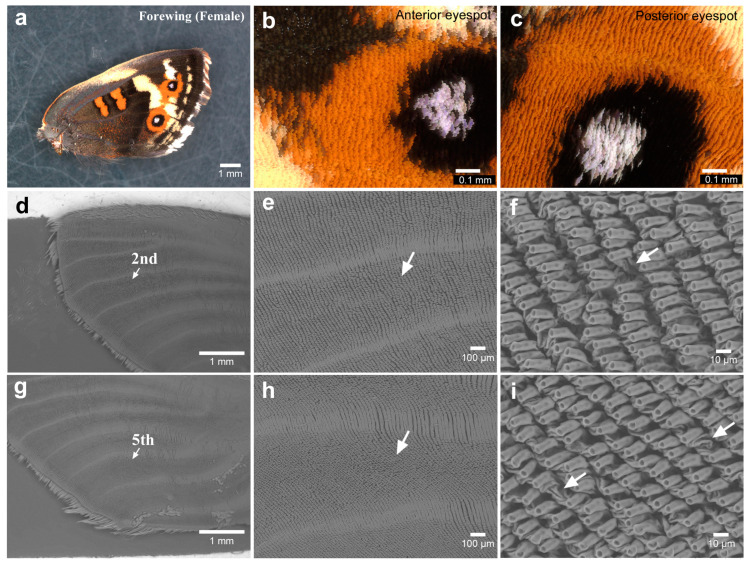
Anterior and posterior eyespots in the unexpanded forewing (female). (**a**) The whole unexpanded forewing. (**b**) Magnification of the anterior eyespot shown in (**a**). (**c**) Magnification of the posterior eyespot shown in (**a**). (**d**) The anterior eyespot without scales. This is a contralateral wing from the wing shown in (**a**–**c**). Panels (**e**–**i**) also show images of the contralateral wing. Thus, the wing margin is to the left. (**e**) Magnification of (**d**). An arrow indicates the focal area, which is nearly at the end of the midline. (**f**) Magnification of (**e**). An arrow indicates a socket interval disorder. (**g**) Posterior eyespot without scales. (**h**) Magnification of the focal area of the posterior eyespot shown in (**g**). An arrow indicates the focal area, which is nearly at the end of the midline. (**i**) Magnification of (**h**). Arrows indicate irregularities in socket intervals.

We next observed the eyespot foci of the unexpanded forewings before eclosion (male *n* = 1; female *n* = 4) (Figure 5a). Microscopy images at the scale level revealed that the scales were vertically packed at high density, and they considerably overwrapped one another (Figure 5b,c). Thus, it was difficult to examine scale arrangement at eyespot foci in such wings, but it may be easier to examine socket arrangement.

We removed scales and made observations by SEM (male *n* = 1; female *n* = 4). Even after removal, the focal area was recognizable nearly at the end of the midline in all individuals examined here (Figure 5d,e). We found that the socket arrangement was irregular at the eyespot foci in the anterior (second) eyespot in four of the individuals examined (Figure 5f). We obtained similar results for the posterior (fifth) eyespot in four individuals examined (Figure 5g–i). 

**Figure 6:**
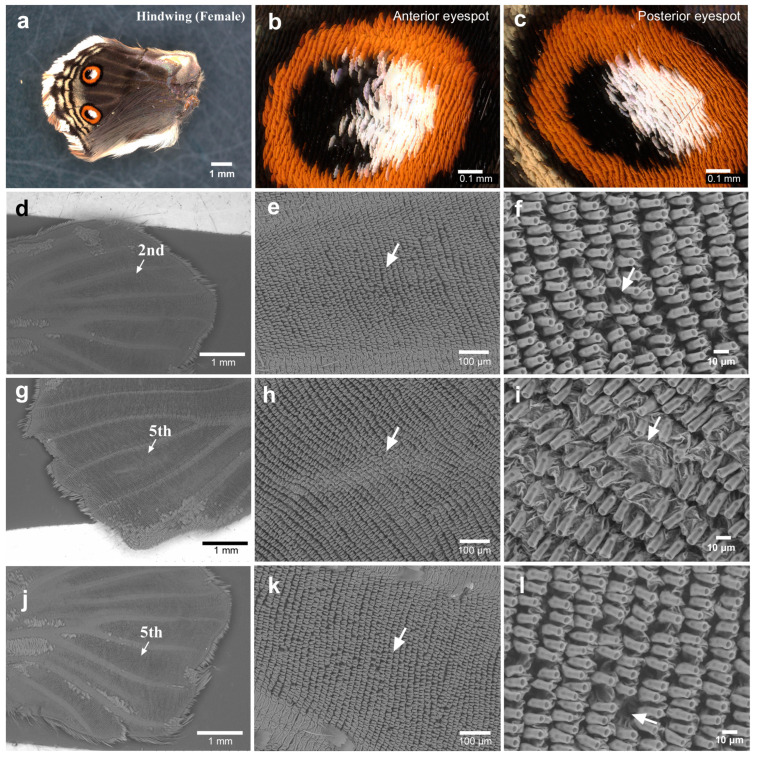
Anterior and posterior eyespots in the unexpanded hindwing (female). (**a**) The whole unexpanded hindwing. (**b**) Magnification of the anterior eyespot shown in (**a**). (**c**) Magnification of the posterior eyespot shown in (**a**). (**d**) The anterior eyespot area without scales. This is a contralateral wing from the wing shown in (**a**–**c**). (**e**) Magnification of (**d**). An arrow indicates the focal area. (**f**) Magnification of (**e**). An arrow indicates a socket interval disorder. (**g**) The posterior eyespot area without scales. This wing is obtained from an individual different from that of (**a**–**f**). (**h**) Magnification of the focal area of the posterior eyespot shown in (**g**). An arrow indicates the focal area, which is nearly at the end of the midline. (**i**) Magnification of (**h**). An arrow indicates disorders of socket intervals. (**j**) The posterior (fifth) eyespot area without scales from yet another individual. (**k**) Magnification of the focal area of the posterior eyespot shown in (**j**). An arrow indicates the focal area. (**l**) Magnification of (**k**). An arrow indicates irregularities in socket intervals.

We also performed similar analyses on the unexpanded hindwings before eclosion. In the light microscopy images, scales were vertically positioned at high density, as in the case of the forewings (male *n* = 1; female *n* = 4) (Figure 6a–c). After the removal of the scales, we made SEM observations (male *n* = 2; female *n* = 4). The focal area in the anterior (second) eyespot was recognizable at the end of the midline in all individuals examined (Figure 6d,e). We observed that the socket intervals were irregular in the focal area in all the individuals examined here (Figure 6f). Similar results were obtained for posterior eyespots in all individuals examined (Figure 6g–l). The posterior eyespots appeared to show more irregularity in the socket intervals than did the anterior eyespots in the hindwing and the anterior and posterior eyespots in the forewing, although this may be a bias from the small sample size.

### 3.3. Potential Focal Areas without Eyespots

**Figure 7:**
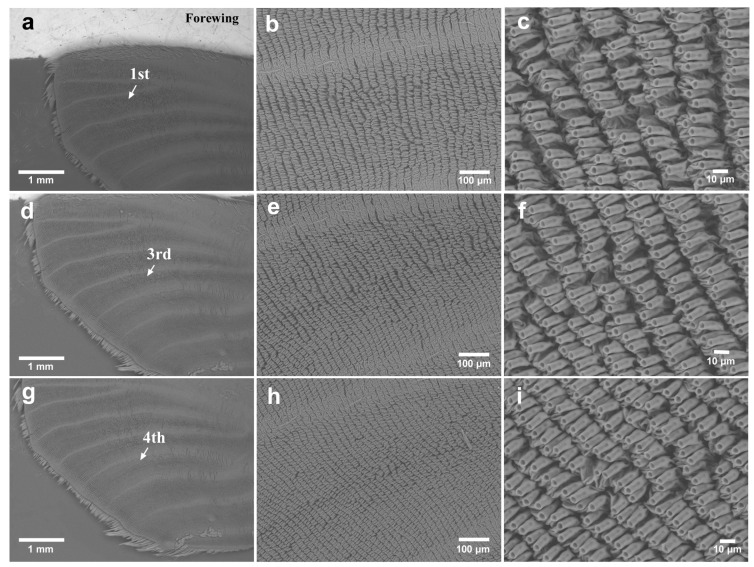
Potential focal areas in the unexpanded forewing. (**a**) The first focal area. (**b**) Magnification of the first focal area shown in (**a**). (**c**) Magnification of (**b**). (**d**) The third focal area. (**e**) Magnification of (**d**). (**f**) Magnification of (**e**). (**g**) The fourth focal area. (**h**) Magnification of the focal area shown in (**g**). (**i**) Magnification of (**h**).

Using SEM, we observed noneyespot focal areas of unexpanded wings before eclosion. In the forewings (male *n* = 1; female *n* = 2), the first focal area showed low levels of irregularities in the socket intervals in all individuals examined (Figure 7a–c). The third focal area and the fourth focal area showed similar socket interval irregularities in all individuals examined (Figure 7d–i). 

**Figure 8:**
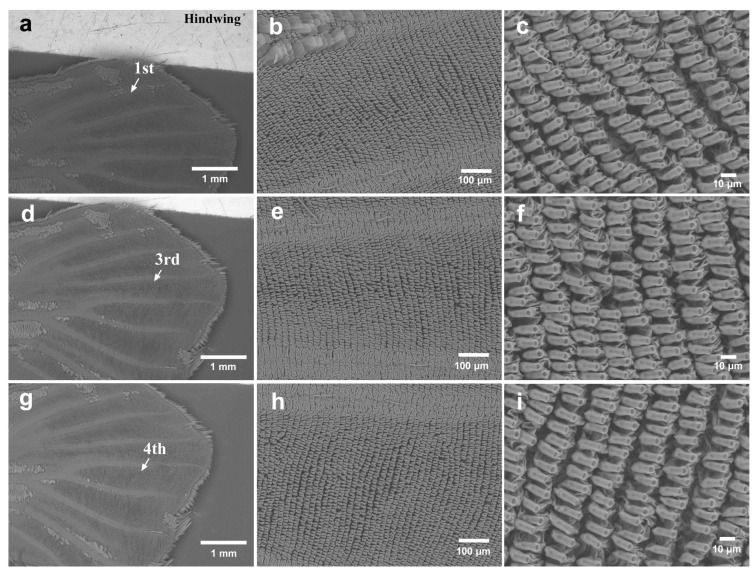
Potential focal areas in the unexpanded hindwing. (**a**) The first focal area. (**b**) Magnification of the first focal area shown in (**a**). (**c**) Magnification of (**b**). (**d**) The third focal area. (**e**) Magnification of (**d**). (**f**) Magnification of (**e**). (**g**) The fourth focal area. (**h**) Magnification of the focal area shown in (**g**). (**i**) Magnification of (**h**).

In the hindwings (male *n* = 1; female *n* = 2), the first focal area showed irregularities between sockets in the two individuals examined (Figure 8a–c). The third and fourth focal areas showed similar socket interval irregularities in the two individuals examined (Figure 8d–i). However, all irregularities in both the forewing and hindwing at the noneyespot focal areas appeared to be relatively mild compared to those at the eyespot focal areas.

### 3.4. Damaged Sites of Expanded Wings after Eclosion

Here, we observed damaged areas in expanded wings after eclosion, focusing on the hindwings. This is because clear ectopic color patterns are often induced in hindwings (which have relatively large background areas) but not in forewings (which do not have large background areas). We hypothesized that there would be disorders in damaged areas similar to those in the eyespot foci because damage-induced color patterns are similar to normal eyespot color patterns. We expected to find disorders of scale arrangement (planar polarity), socket array irregularities, and wing membrane distortions as detected in the natural eyespot foci in the previous sections.

**Figure 9:**
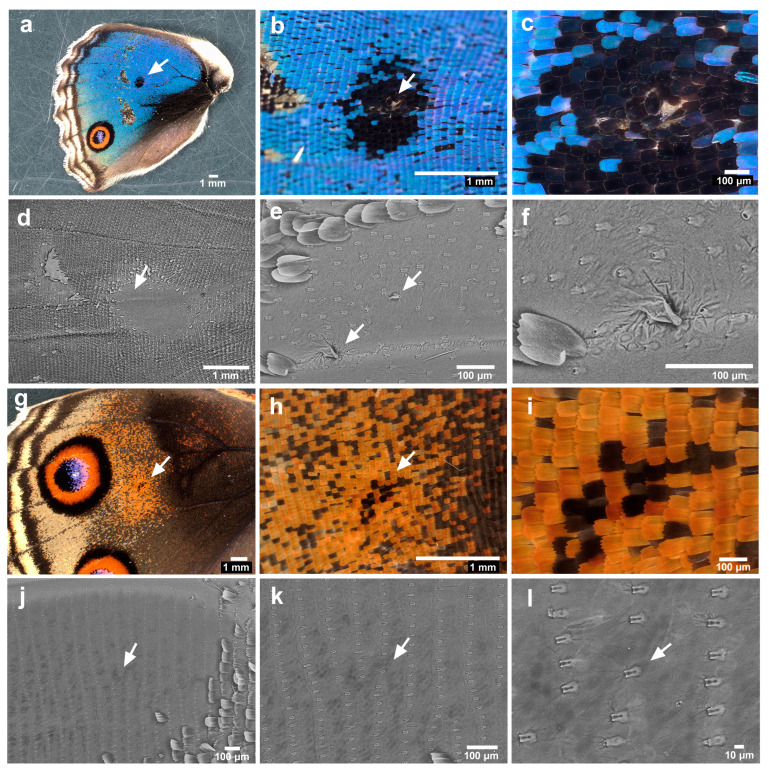
Damage-induced hindwing color patterns. (**a**) A whole male wing with a damage-induced black spot. An arrow indicates the induced spot. (**b**) Magnification of the damage-induced black spot shown in (**a**). An arrow indicates the damaged point. (**c**) Magnification of (**b**). Scales are disordered in direction and density. (**d**) Damaged area without scales. The damaged area is indicated by an arrow. (**e**) Magnification of (**d**). Arrows indicate socket irregularities in the direction and substantial distortions of the wing membrane. (**f**) Magnification of (**e**). (**g**) A wing with a damage-induced orange area (an arrow). (**h**) Magnification of (**g**). There are black scales at the center (an arrow). (**i**) Magnification of (**h**). (**j**) A damaged area without scales. The arrow indicates the area of socket array irregularities. (**k**) Magnification of (**j**). An arrow indicates the area of socket array irregularity. (**l**) Magnification of (**k**). An arrow indicates an isolated socket with large intervals.

We first used a light microscope (male, *n* = 4; female, *n* = 4). In one male individual, physical damage to the pupal wing tissue immediately after pupation induced an ectopic black spot (Figure 9a,b). The scales were disrupted with irregular directions (planar polarity) and sizes (Figure 9c). We also obtained SEM images (male *n* = 4; female *n* = 3). After removing the scales from this individual (Figure 9d), we observed striking distortions of the wing membrane using SEM (Figure 9e,f), which were reminiscent of the wing membrane distortions detected in normal eyespot focal areas (Figure 3l and Figure 4g,h).

In another female individual, physical damage to the pupal wing tissue immediately after pupation induced dark orange and black scales with no disorder of scale arrangement (planar polarity) (Figure 9g–i). After removing the scales from this individual (Figure 9j), extensive irregularities were not observed via SEM (Figure 9k). However, a socket, likely located at the center of the damaged zone, was associated with long socket intervals (Figure 9l). In total, we observed scale arrangement (planar polarity) disorders as above in three females and three males in light microscope images among the individuals examined (male, *n* = 4; female, *n* = 4). In the SEM images, three male individuals and two female individuals showed similar socket array irregularities among the individuals examined (male *n* = 4; female *n* = 3).

### 3.5. Damaged Sites of Unexpanded Wings before Eclosion

**Figure 10:**
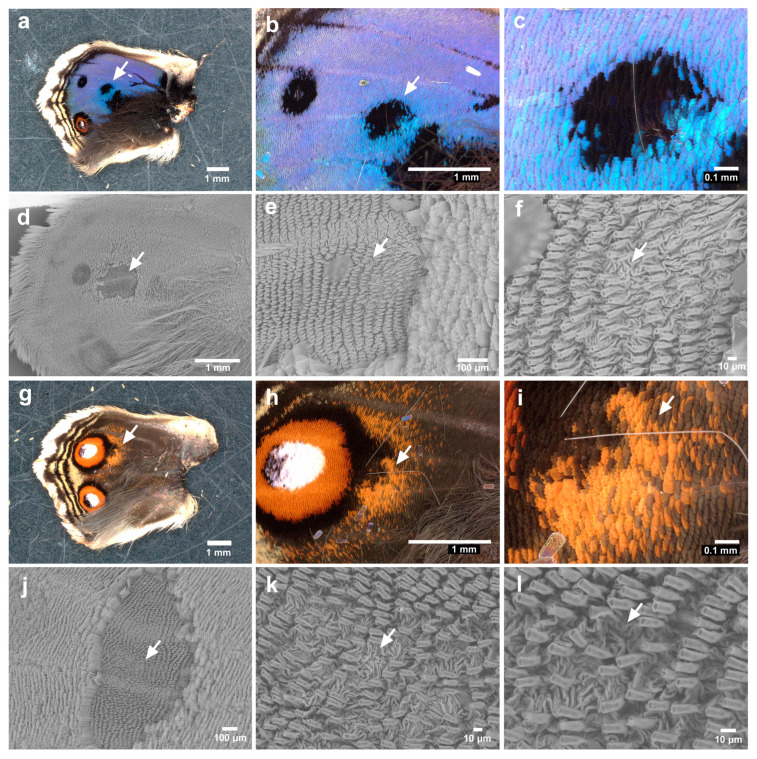
Damage-induced color patterns in the unexpanded hindwing. (**a**) A whole male wing with a damage-induced black spot. An arrow indicates the induced spot. (**b**) Magnification of the damage-induced black spot shown in (**a**). (**c**) Magnification of (**b**). (**d**) Damaged area without scales. The damaged area is indicated by an arrow. (**e**) Magnification of (**d**). An arrow indicates the damaged area of the wing membrane. (**f**) Magnification of (**e**). There are many socket array irregularities (an arrow). (**g**) Wing with a damage-induced orange area (an arrow). (**h**) Magnification of (**g**). An arrow indicates the damaged area. (**i**) Magnification of (**h**). An arrow indicates the damaged area. (**j**) A damaged area without scales. An arrow indicates the area of socket array irregularities. (**k**) Magnification of (**j**). An arrow indicates the area of socket array irregularities. (**l**) Magnification of (**k**). An arrow indicates the irregular socket intervals.

We next observed damaged hindwing sites on the unexpanded wings before eclosion (male *n* = 3; female *n* = 3). In one individual (male), a damage-induced black spot was observed (Figure 10a–c). Because of the high density of scales, scale arrangements could not be examined well (Figure 10c). We thus removed the scales for SEM to observe the socket arrangements (male *n* = 3; female *n* = 3) (Figure 10d). There seemed to be a disordered area (Figure 10e), in which the socket intervals were severely disrupted (Figure 10f). The exposed socket intervals revealed numerous irregular folds of the wing membrane.

In another individual (female), physical damage induced orange scales with disruption of the outer black ring of the anterior (second) eyespot (Figure 10g–i). We removed the scales for SEM (Figure 10j), which revealed that the socket intervals and directions (planar polarity) were severely disrupted (Figure 10k,l). In all other individuals, similar disorders of various degrees were found. These disorders might be considered more severe than those of natural eyespots.

### 3.6. Relationships among Scales, Sockets, and Wing Membrane

To further observe scale arrangement disorders, socket array irregularities, and wing membrane distortions and to understand their relationships, we examined the female anterior eyespots (*n* = 60), the female posterior eyespots (*n* = 60), and the male posterior eyespots (*n* = 44) in hindwings with a light microscope. According to the working definition of the scale arrangement disorder (see Materials and Methods), we found 23 (38%), 23 (38%), and 14 (32%) individuals that showed the disorder in the female anterior eyespots, the female posterior eyespots, and the male posterior eyespots, respectively, based on the eyespot focal images. These percentages revealed that the scale array disorder was not rare among the individuals examined, although the identification of the disorder was not free from the bias of researchers.

**Figure 11:**
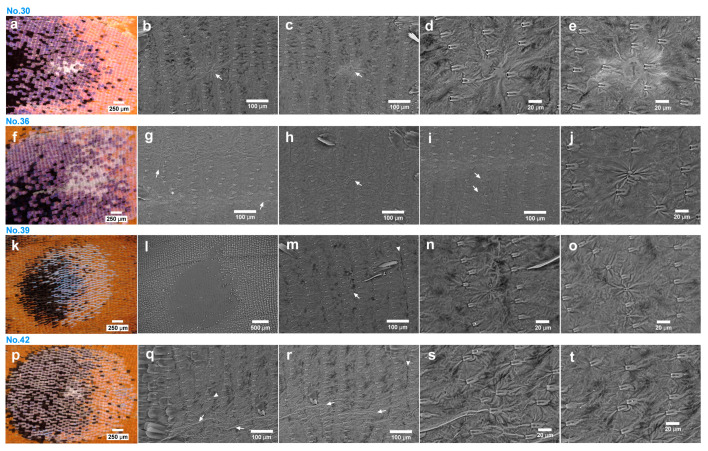
Female anterior eyespot focal areas of the nonrandom sampling group via light microscopy and SEM. Four representative samples are shown. Each line of panels contains images of a single individual. (**a**) Eyespot focal area of individual No. 30. (**b**,**c**) Wing membrane distortions at a branching point of socket arrays (arrows). (**d**) Magnification of (**b**). (**e**) Magnification of (**c**). (**f**) Eyespot focal area of individual No. 36. (**g**) Socket arrays with many branching points. Arrows indicate ridges or furrows parallel to socket arrays. (**h**) Socket arrays with irregularities and wing membrane distortion (an arrow). (**i**) Socket arrays with displaced sockets (arrows). (**j**) Magnification of (**h**). (**k**) Eyespot focal area of individual No. 39. (**l**) Socket arrays with many branches. (**m**) A displaced socket with wing membrane distortion (an arrow). (**n**) Magnifiction of (**m**). (**o**) A socket with the wing membrane distortion at a branching point of socket arrays. (**p**) Eyespot focal area of individual No. 42. (**q**) Socket arrays with an irregular socket at a branching point (an arrowhead). Ridges perpendicular to socket arrays are indicated (arrows). (**r**) Socket arrays with perpendicular ridges (arrows) and a parallel furrow (an arrowhead). (**s**,**t**) Magnification of q.

We then focused on the female anterior eyespot to clarify the frequency of socket array irregularities and wing membrane distortions to obtain more information on their structures via SEM. We here selected 11 female samples (from a pool of 60) with a severe scale arrangement disorder in the anterior eyespot (nonrandom sampling group: Individual ID No. 1, 3, 10, 11, 14, 21, 30, 36, 39, 42, and 48). All samples showed extensive socket array irregularities. Branching (or fusion) of socket arrays was frequently found with a few displaced sockets at a branching point (Figure 11). Thus, the frequency of the socket array irregularities was 100%. On the other hand, only four individuals showed wing membrane distortions (Individual ID No. 30, 36, 39, 42; Figure 11a–t); the frequency was 36%. They varied structurally, but some of them were associated with multiple sockets, and some were present between socket arrays (Figure 11). We also discovered furrows (or ridges) running parallel to socket arrays and ridges (or furrows) running perpendicular to socket arrays (Figure 11g,m,q,r). 

**Figure 12:**
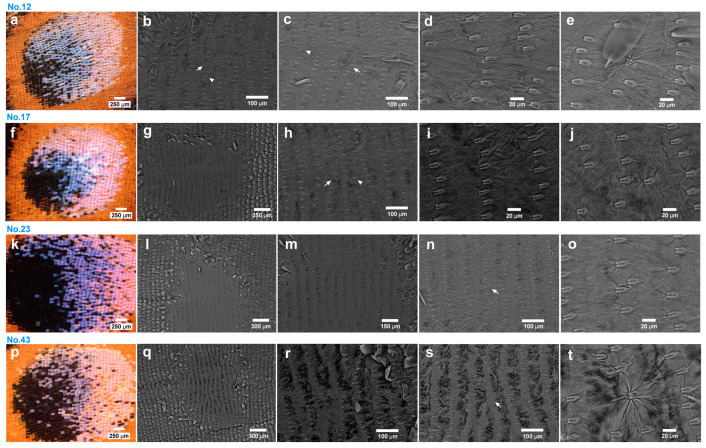
Female anterior eyespot focal areas of the random sampling group via light microscopy and SEM. Four representative samples are shown. Each line of panels contains images of a single individual. (**a**) Eyespot focal area of individual No. 12. (**b**) Socket arrays with irregularities. Long (an arrow) and short (an arrowhead) socket intervals are indicated. (**c**) Socket arrays with an extra socket (an arrow) and wing membrane distortion (an arrowhead). (**d**,**e**) Magnification of (**c**). (**f**) Eyespot focal area of individual No. 17. (**g**) Socket arrays with many branching points. (**h**) Socket arrays with a long socket interval (an arrow) and a displaced socket at a branching point (an arrowhead). (**i**) Magnification of (**h**) (left side). (**j**) Magnification of (**h**). (**k**) Eyespot focal area of individual No. 23. (**l**) Socket arrays with a relatively small number of branching points. (**m**) Magnification of (**l**). (**n**) Socket array with an irregular socket (an arrow). (**o**) Magnification of (**n**). (**p**) Eyespot focal area of individual No. 43. (**q**) Socket arrays with branching points. (**r**) Magnification of (**q**). (**s**) Magnification of (**q**). A displaced socket at a branching point is indicated by an arrow. (**t**) Magnification of (**s**). An isolated large socket is shown.

We performed the same observations in 11 female samples (from a pool of 60) of the random sampling group (Individual ID No. 9, 12, 17, 21, 23, 43, 46, 47, 48, 51, and 54). In this random group, we again found socket array irregularities in every individual with various degrees (Figure 12), although branching may not be as extensive as in the nonrandom group. Hence, the frequency of the socket array irregularities was 100%. On the other hand, we found just one individual that had wing membrane distortion (Individual ID No. 42; Figure 12p–t). Hence, the distortion rate was 9%. These two groups were not significantly different statistically in terms of frequencies of the wing membrane distortion (*p* = 0.31; Fisher’s exact test), suggesting that the scale arrangement disorder was not a good indicator for the wing membrane distortion. Additionally, we discovered an isolated large socket (Figure 12s,t).

## 4. Discussion

### 4.1. Socket Array Irregularities and Wing Membrane Distortions

In this study, we observed scale arrangement disorder at eyespot foci via a light microscope, and we discovered socket array irregularities and physical distortions of the wing membrane at eyespot foci (collectively called focal disorders) via SEM in the Blue Pansy butterfly *J. orithya*. We examined the anterior and posterior eyespots both in the forewings and hindwings. Overall, the hindwings were more severely disordered than the forewings. This result may be simply because larger eyespots tend to make these disorders more severe than smaller eyespots. It is likely that eyespots without such disorders may be “repaired” successfully as a normal developmental process. Indeed, the disorders were not always found among the individuals examined, even in relatively large eyespots. This line of discussion is also consistent with the observation that the presumptive foci that do not form eyespots have only minor irregularities in the socket intervals.

One may argue that focal disorders may be developmental anomalies that are not related to critical developmental processes. If so, the present discovery of focal disorders is considered trivial. We disagree. Eyespot focus has a distinct decoration called a pupal cuticle focal spot (or simply cuticle spot) on the pupal surface, and a pupal cuticle focal spot is sometimes associated with a cuticle mark [13]. Pupal cuticle focal spot size is correlated with adult eyespot size in *Junonia* and is conserved among many nymphalids and other butterfly species [55]. We speculate that the pupal cuticle focal spot has an important function as a mechanical base for organizing cells to receive mechanical signals. Thus, the focal disorders detected in the present study are probably produced as a consequence of mechanical signal reception at organizing cells. Alternatively, focal disorders may be produced as a consequence of the mechanical activity of organizing cells.

A scale arrangement disorder in our definition was not rare at the eyespot focal areas; it can be found in more than 30% of the individuals at the anterior eyespot of the hindwing (Figure 11), although it was 10% at the posterior eyespot of the hindwing (Figure 4). It appears that an eyespot focus always has socket array irregularities, which are often associated with branching points of socket arrays. Socket array branching may be an important feature of an eyespot focus. A wing membrane disorder was found in 9% of the random sampling group and in 36% of the nonrandom group (Figure 11 and Figure 12), although their frequencies are not significantly different.

Equally important, a scale arrangement disorder was found basically in a focal area containing white or blue structural focal scales. The white structural focal scales may be scales produced by organizing cells (assuming that organizing cells may differentiate into scale-building cells with no clear qualitative difference in cellular and subcellular structures [52]). If so, scale-building cells from organizing cells will likely produce large scales. We found a large socket in this study, but it was in isolation and not clustered (Figure 12s,t). Certainly, we need more anatomical information on organizing cells and their products in the future.

In general, an eyespot can be produced perfectly without white focal scales [75]. Moreover, the white structural scales can be positioned outside an eyespot body in *Calisto tasajera* (Nymphalidae: Satyrinae) [75]. Such semi-independent behaviors of the white scales or other sub-elements are called the uncoupling rule [58,75]. Probably, eyespot organizing cells may produce either normal pigment scales or white structural scales depending on mechanical tension from surrounding cells (i.e., from the previous organizers in a serial induction process).

Likely because the whole wing is not square in shape, socket arrays must occasionally have branches, fusions, or irregular shapes to cover the entire circular wing area [76]. Although we have not thoroughly examined this phenomenon, a scale disorder is unlikely to be frequent throughout a wing outside the eyespot focal area in butterflies in general; almost always, butterfly wing scales are well arranged in equal intervals in rows [1,76]. Thus, it is likely that not only scale arrangement disorders but also socket array irregularities and wing membrane distortions outside an eyespot focus are not as frequent as those at an eyespot focus. However, we do not claim that the disorders found in the eyespot focal areas are specific to eyespots. Other color pattern elements may have similar disorders, especially when white or blue structural scales are present. Interestingly, a large scale was reported at a chiasmatic branching point of scale (socket) arrays in *J. orithya* [76]. Its relation to focal disorders may be of interest in the future.

### 4.2. Mechanical Signals and Mechanical Organizers

There may be a few different types of socket array irregularities. First, the socket intervals were shorter or larger than the surrounding regular intervals. In this case, the sockets are still on the regular lines of the arrays. Second, the position of the sockets was displaced from the regular arrays. Third, the direction (planar polarity) of the sockets was affected. This third type is likely caused mostly by wing membrane distortions. Indeed, the position and planar polarity of sockets were severely affected in the areas where the wing membrane was severely distorted. It appeared that many of the socket array irregularities occurred at branching points of socket arrays. 

**Figure 13:**
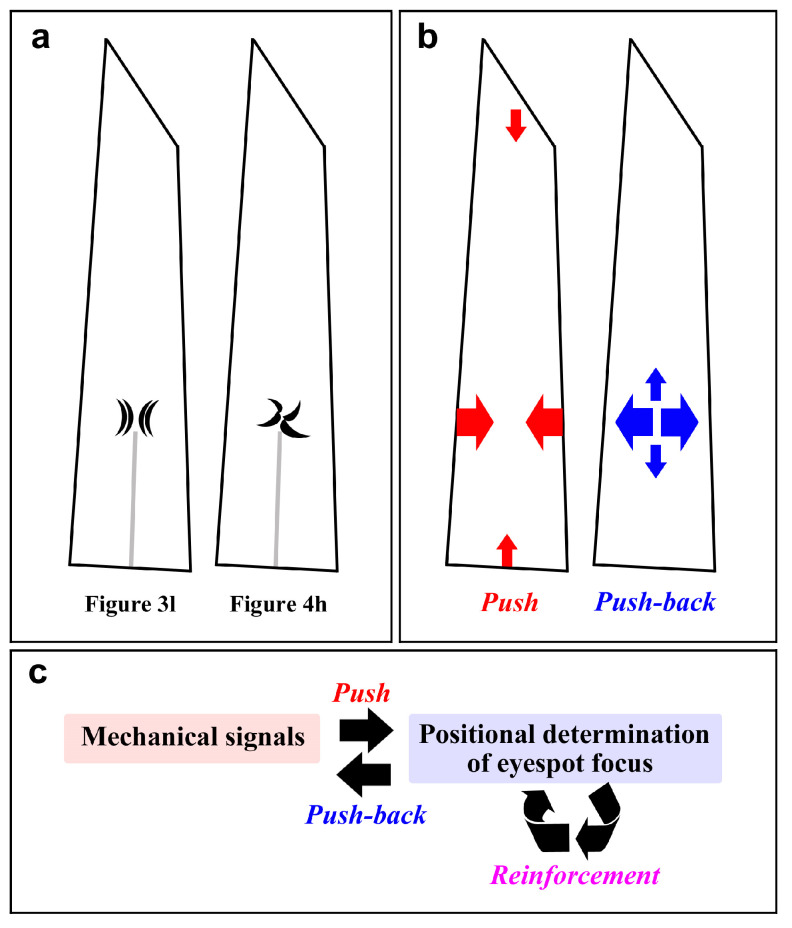
Physical distortions and mechanical signals. (**a**) Illustration of the physical distortions of the wing membrane detected in this study. The distortion is located at the tip of the midline. (**b**) Possible mechanical forces from the wing veins, the marginal band organizer, and the discal organizer (left). The prospective eyespot organizer then pushes back to balance forces (right). (**c**) Feedback and reinforcement of the mechanical signals from the prospective eyespot organizer. Reinforcement indicates further secretion of the cuticle to bind to the pupal cuticle focal spot more tightly and the polyploidization of organizing cells.

The wing membrane is certainly affected by individual sockets and their associated membrane locally, but importantly, at least some wing membrane distortions appear to express nonlocal mechanical pushes from both sides where the wing veins are located (Figure 3, Figure 11 and Figure 12). A type of distortion (Figure 3k and Figure 11d) might have been produced by bi-sided squeezing forces. Long ridges and furrows parallel and perpendicular to socket arrays (Figure 11g,q–s) might have been produced by forces from two directions. Somewhat vortex-like distortions (Figure 4h) might have been produced due to strong pushes and an imbalance of the forces from both sides. Based mainly on these wing membrane distortions (Figure 13a), possible mechanical signals are shown in Figure 13b. Mechanical signals may originate not only from the wing veins (the wing vein organizers) but also from the wing margin (the organizer of the marginal band system) [77] and from the wing basal area (the organizer of the discal symmetry system) [6]. The squeezed cells must push back to achieve mechanical balance; the organizing cells may more tightly attach to the thickened cuticle that forms a cuticle spot [13,55]. The binding of cells to the cuticle is important for the transduction of mechanical morphogenic signals [66,67,68]. As a result, the organizing cells further “accumulate” mechanical signals at the hardest place (the cuticle spot), which is resistant to pushes. Organizing cells may also become larger in size by polyploidization to push them back [53,54]. This reinforcement of the organizing cells gradually solidifies the position of the eyespot organizer (Figure 13c). The extra push-backs may then produce secondary organizers [6,58]. These interpretations are consistent with the physical distortion hypothesis, which states that distortion waves (mechanical stress) on the pupal wing sheet from organizers function as a morphogenic signal (mechanical morphogen) to initiate serial induction of organizers for color pattern determination in butterfly wings [58].

Wing veins are important structures that contribute to color patterns [1,6,78]. As discussed above, the focal cells may be squeezed by the force from wing veins located on both sides of the focal cells. The low-frequency contraction–relaxation movements of the pupal wing tissues may contribute to this process [49,50,51,79]. The color pattern determination process occurs under dynamic tissue movement in butterflies [79]. Notably, we also observed the midline as a prominent band or groove in the wing compartment and that the eyespot focal area seemed to be located at the proximal edge of this band as if both the midline and the eyespot focal area were the products of a mechanical squeeze. This may be why an eyespot is always located in the midline, which is called the midline rule [58]. The formation of the midlines and the placement of eyespot foci at the tips of the midlines coincide with *Distal-less* expression [35,80]. These mechanical signals must occur before apolysis, i.e., before the wing tissue is detached from the opposing cuticle.

### 4.3. Unexpanded Wings and Damage-Induced Modifications

In addition to examining the expanded adult wings, we also examined the unexpanded wings to determine whether the focal disorders observed in expanded wings can also be observed in unexpanded wings. Unexpanded wings had a higher density of scales and sockets than expanded wings [81], but socket intervals were easily discernible in unexpected wings. Socket array irregularities (uneven intervals) in the unexpanded wings were confirmed, suggesting that they are not due to a failure of the wing expansion process per se during eclosion but rather due to an inherent developmental process that occurred within the pupal tissue before expansion. On the other hand, we could not directly observe wing membrane distortion in unexpanded wings because the wing membrane is folded like a pleated dress [81]. It was difficult to detect structural evidence for mechanical squeezing forces in a folded sheet. However, it should be noted that the socket array irregularities are tightly linked to the folding of the wing membrane.

Given that socket array irregularities and wing membrane distortions occur in pupal wings during the process of natural development, an important question is when they occur during development. Because physical damage at the early pupal stage results in socket array irregularities and wing membrane distortions, focal disorders in nondamaged natural wings may also be determined at the early pupal stage before or during the color pattern determination, as in damaged wings. Although at that point socket arrays and the wing membrane are not yet produced, the contraction–relaxation movement of the wing tissue continues to the period of the wing membrane formation when mechanically induced disorders are recorded in the wing membrane.

Although the effect of physical damage varied, chaotic socket irregularities and wing membrane distortions were observed. These observations were similar to those observed in natural eyespot foci but may be more severe. The microstructural similarities between the damaged sites and the natural eyespot foci suggest that mechanisms for color pattern formation are similar between the two systems. Physical damage likely causes tremendous tissue destruction in damaged areas, including cell death and tissue deformation. To repair such a damaged site, hemocytes may quickly aggregate, and surrounding epithelial (epidermal) cells must invade the damaged site and secrete cuticle. The damaged site may serve as a force accumulation site because the damaged site must be solidified by cuticle secretion for the wing membrane, which may function as an induced pupal cuticle focal spot. These activities may be somewhat similar to those of natural eyespot foci. In other words, a process similar to physical damage repair may occur in natural eyespots [27].

### 4.4. Conventional Models and Mechanical Model

Conventional morphogens (i.e., molecular morphogens) for biological morphogenesis are thought to travel by diffusion in accordance with a gradient model. In general, gradient models or reaction–diffusion (RD) models have been used to explain biological color pattern formation. In gradient models, morphogens should travel long distances within a limited critical period, and stable gradients should be established, which are not easy to achieve in butterflies [56,57]. RD models may be suitable for probabilistic color patterns, such as those found in fish and mammals, but not for deterministic ones, such as butterfly wing color patterns. It appears that a robust model is not easily obtained to explain color pattern rules and the reproducible anatomical positions of color pattern elements in butterfly wings [56,57,58,80], but the extent to which the RD process is responsible for the eyespot determination process should be investigated further because some studies have reported successful simulations of eyespot formation via RD models [1,35,80]. Among them, the grass-fire model is a non-Turing-type RD model and can explain both the eyespot and its associated parfocal element in response to temperature-induced modifications [80]. Remarkably, this model is reportedly scale-free. Butterfly color patterns are known to scale proportionally among individuals of various sizes [82]. This is understandable because butterfly color pattern elements are two-dimensional anatomical structures but not simple “paintings” [1]. In this sense, the grass-fire model appears to have some truth, and it is probably the best mathematically simulated model thus far (note that the induction model has not been mathematically simulated in detail except for the application of Newtonian equations). On the other hand, the scaling problem may be explained relatively easily by the physical distortion hypothesis because mechanical signals are scalable as long as the force-mediating medium is solid enough to transduce mechanical signals. 

In the grass–fire model [80], the signal is the moving wavefront from the wing veins. This aspect is similar to the physical distortion hypothesis. In the grass-fire model, the wavefront is not stabilized, so how to “read” the proper wavefront is a problem associated with the grass-fire model. Once the wave front passes, the “fuel” is consumed. Thus, the system should precisely determine the position of the organizer via a single signaling event. In contrast, no “fuel” is consumed in the mechanical model because the signals are mechanical. Thus, mechanical signals can be repeatedly released until the task is completed. It should be noted that mechanical power can travel instantly in a solid medium in a scale-free manner. A steady state similar to that of the gradient and RD models is also unnecessary for the mechanical model, except for the force balance. Furthermore, in the mechanical model, there is no problem associated with how to read the proper wavefront.

The butterfly wing size and shape appear to be determined by the cell size and cell numbers [76,82]. To do so, wing epithelial (epidermal) cells may sense the mechanical signals of neighboring cells. Mechanosensory channels may play an important role in mechanical signal transduction in butterfly wings [58]. At this point, we do not know whether the hypothetical mechanical signals are identical to the mechanical forces that drive the contraction–relaxation movement of the tissue [49,50,51,79]. In any case, color pattern determination in butterfly wings does not occur in static tissue. Instead, it occurs in mechanically dynamic tissue [49,50,51,79], which has been ignored in developmental models. Despite this physical stress, butterfly eyespot morphogenesis must be robust at various physical scales (size) [82]. 

It is worth noting that the idea of mechanical signals in development is not new. For example, *Xenopus* gastrula embryos appear to use mechanical tension as a developmental cue [83]. Similarly, *Xenopus* notochord morphogenesis involves osmotic pressure [84]. A recent study showed that mechanical manipulations induce the formation of additional somites in chick embryos [85]. Many studies have suggested that mechanical signals are instructive in development [86,87,88,89,90,91,92,93,94,95]. Biological tissues may behave like a liquid crystal, and morphogenesis, including regeneration of *Hydra*, may be explained by its mechanics [96,97,98,99,100,101,102,103,104]. The butterfly wing system may be an important system for visualizing mechanical disorders in an organism under natural and experimental conditions.

## 5. Conclusions

The present study revealed that scale arrangement disorders, socket array irregularities, and wing membrane distortions were present at eyespot foci in butterfly wings. Damage-induced ectopic eyespots are also associated with similar disorders. These focal disorders may be caused by mechanical forces from surrounding cells or previous organizers that determine the position of the focal organizer. Alternatively, disorders may signify the activities of organizing cells at foci. These results are consistent with the distortion hypothesis for color pattern determination in butterfly wings, in which mechanical signals are used in the serial induction of organizers. Broadly speaking, the mechanical signals here in butterfly wings may be closely related to the differentiation waves explained in seminal books and papers [105,106,107,108]. In the future, we expect further SEM research to characterize the microstructures of sockets and wing membranes, including branching of socket arrays, ridges, furrows, large scales, and midlines, in addition to socket array irregularities and wing membrane distortions. Fine images of the pupal wing tissue at the cellular and subcellular levels using transmission electron microscopy (TEM) may be helpful in clarifying the microstructures of organizing cells and the associated cuticle spots. We also expect time-lapse movies that show possible mechanical changes in the pupal wing tissue and cells.

## Data Availability

This paper contains all relevant data obtained in this study.
